# Clinical Outcomes Following the Implementation of a Novel One-Year Training Program in Emergency Medicine in Karachi, Pakistan

**DOI:** 10.5334/aogh.3890

**Published:** 2023-01-19

**Authors:** Syed Ghazanfar Saleem, Saima Ali, Adeel Khatri, Sama Mukhtar, Wasfa Farooq, Quratulain Maroof, Muhammad Imran Jamal, Tariq Aziz, Kaniz Farwa Haider, Farah Z. Dadabhoy, Megan M. Rybarczyk

**Affiliations:** 1 Emergency Services, Indus Hospital and Health Network, Karachi, Pakistan; 2 Pediatric Oncology, Indus Hospital and Health Network, Karachi, Pakistan; 3 Gerald R. Ford School of Public Policy, University of Michigan, USA; 4 Department of Emergency Medicine, Harvard Medical School, Brigham and Women’s Hospital, Boston, MA, USA; 5 Department of Emergency Medicine, Perelman School of Medicine, University of Pennsylvania, Philadelphia, PA, USA

**Keywords:** emergency care, emergency medicine, training, education, clinical outcomes

## Abstract

**Background:**

Most Emergency Departments (EDs) in low- and middle-income countries (LMICs), particularly in Pakistan, are staffed by physicians not formally trained in Emergency Medicine (EM). As of January 2022, there were only 13 residency training programs in EM throughout all of Pakistan. Therefore, an intermediate solution—a one-year training program in EM—was developed to build capacity.

**Objective:**

To determine the impact of a novel training program in EM on clinical metrics and outcomes.

**Methods:**

The first cohort of a novel, one-year training program—the Certification Program in Emergency Medicine (CPEM)—completed the program in June 2019. The program consisted of two arms: CPEM-Clinical (CPEM-C), which included physicians from the Indus Hospital and Health Network (IHHN) ED; and CPEM-Didactic (CPEM-D), which included physicians from EDs across Karachi. Both groups participated in weekly conferences, such as didactics, small group discussions, workshops, and journal clubs. CPEM-C learners also received clinical mentorship from local and international faculty. Mortality, length of stay (LOS), and time-to-evaluation, as well as metrics in four key areas—patients at risk for cardiovascular disease/acute coronary syndrome, sepsis, respiratory illness, and intra-abdominal trauma—were assessed before and after the initial cohort at IHHN and compared with other groups in IHHN.

**Findings and Conclusions:**

More than 125,000 patients were seen from July to December 2017 (pre-CPEM) and July to December 2019 (post-CPEM). Overall, there were significant improvements in all clinical metrics and outcomes, with the exception of LOS and time-to-evaluation, and a trend toward improved mortality. In comparing CPEM graduates to other groups in IHHN ED, most metrics and outcomes significantly improved or trended toward improvement, including mortality. Implementation of a medium-duration, intensive EM training program can help improve patient care and the development of EM as a new specialty in lower-resource settings.

## Introduction

Emergency care systems are an important part of functioning healthcare systems, particularly in low-resource settings. In fact, the recent spread of diseases such as Ebola and SARS-CoV-2 have revealed weaknesses even in health care systems that had been previously considered to be strong and have highlighted the imperative for emergency care. In low- and middle-incomes countries (LMICs) in particular, it has been suggested that more than half of deaths could be avoided with functioning emergency care systems [1].

At the World Health Assembly in May 2019, the World Health Organization (WHO) passed an important resolution—WHA 72.16: Emergency Care Systems for Universal Health Coverage: Ensuring Timely Care for the Acutely Ill and Injured—which calls even more attention to the current lack of and the need for high-functioning emergency care systems, particularly in LMICs [2]. Additionally, the third, and most recent, edition of Disease Control Priorities (DCP-3) now includes a chapter on strategies to strengthen health care systems to provide emergency care, including prioritizing standardization of protocols and training [3]. Attention to this vital part of health care systems is growing; however, investment is now needed in both training and infrastructure in order to actually develop these systems, particularly in LMICs.

As a developing country with a tremendously growing population, an under-resourced primary health care system, an increasing rate of fatal injuries, and with nearly the lowest percent of its gross domestic product (GDP) spent on health (just 3.2% as of 2018) in the region, Pakistan has immense need of a robust and efficient emergency care system and workforce [4,5]. Studies show that emergency conditions, such as injuries and complications from cardiovascular diseases, are among the leading causes of morbidity and mortality [5,6]. Furthermore, inaccurate diagnosis by first level providers and improper triage from first level facilities are the some of the main causes of poor outcomes of many surgically treatable illnesses [7].

In Pakistan, it has been estimated that approximately 15,000 to 30,000 trained emergency physicians are needed to serve the population of more than 200 million [8]. In 2011, the College of Physician and Surgeons Pakistan (CPSP) started a formal five-year training program in emergency medicine (EM). As of the beginning of 2022, only 13 institutes had initiated formal training programs. As a result, at present, most emergency departments (EDs) in Pakistan are staffed by physicians not formally trained in EM, which can result in poor patient outcomes. Given the small number of EM programs currently running in Pakistan, it will take decades to train enough physicians to match the needs of this ever-growing population.

In light of the above-mentioned circumstances, a novel, intensive, medium-duration program of modular training and certification was developed to provide physicians with additional training in the necessary knowledge and skills to fill this gap and to provide emergency care more effectively in Pakistan—the Certification Program in Emergency Medicine (CPEM).

The one-year Certification Program in Emergency Medicine (CPEM) was developed by specialists from IHHN in Karachi, Pakistan and Brigham and Women’s Hospital (BWH), a teaching affiliate of Harvard Medical School in Boston, Massachusetts in the US between 2017 and 2018. The program was launched in July 2018 at IHHN; the first cohort completed their training in June 2019 and demonstrated improved learning outcomes [16]. Of note, the program graduated its third cohort of trainees July 2022 with an adapted, mostly virtual curriculum due to the ongoing SARS-CoV-2 pandemic as well as an expanded cohort in Bangladesh. Core faculty from the program are currently at the Perelman School of Medicine at the University of Pennsylvania and IHHN.

The twelve-month, twelve-block training program was designed to provide fundamental knowledge and supervised clinical experience in the provision of emergency care. Each block progresses from knowledge acquisition to application (Table 1). Local and international EM faculty developed, adapted, and administered the training materials, using a combination of live instruction, small group sessions, skills workshops (including procedures and point-of-care ultrasound) and simulation.

**Table 1:** Block schedule for the 2018–19 CPEM academic year. *ACLS: Advanced Cardiac Life Support; ECG: electrocardiogram; ATLS: Advanced Trauma Life Support; FAST: Focused Assessment with Sonography in Trauma; PALS: Pediatric Advanced Life Support; NIHSS: National Institutes of Health Stroke Scale*.


MONTH	TOPIC	KEY SKILLS AND PROCEDURES

July	Cardiovascular	ACLS, echocardiogram, ECG, pericardiocentesis, central venous catheter placement, ultrasound-guided intravenous line placement, ankle-brachial indices, pulsus paradoxus

August	Pulmonary	Intubation, mechanical ventilation, non-invasive positive pressure ventilation, arterial blood gas, chest tube/needle decompression, thoracentesis, lung ultrasound

September	Trauma	ATLS, FAST/e-FAST

October	Orthopedics, Immunology, Rheumatology, Dermatology	Arthrocentesis, laceration repair, wound care, incision and drainage, procedural sedation, nerve blocks, splinting, joint reduction, soft tissue/musculoskeletal ultrasound, x-ray interpretation

November	Renal, Genitourinary, Gynecology	Foley placement, renal/bladder/pelvic ultrasound, lab interpretation (electrolytes)

December	Obstetrics, Pediatrics	PALS, emergency delivery, intraosseous line placement, pediatric lumbar puncture, pediatric intravenous access, obstetrical and pediatric ultrasound, pediatric x-ray interpretation

January	Gastroenterology	Nasogastric tube placement, paracentesis, abdominal ultrasound

February	Neurology	NIHSS, lumbar puncture

March	Ophthalmology, Otorhinolaryngology	Slit lamp exam, foreign body removal, peritonsillar abscess drainage, nasal packing, lateral canthotomy, dental block, eye ultrasound

April	Hematology, Oncology, Endocrinology	Lab interpretation (hematology and coagulation studies, endocrinology studies)

May	Psychiatry, Toxicology	Pharmacology, ECG interpretation

June	Infectious Diseases	Antibiotic stewardship, review of ultrasound and procedures


Thirty-two physicians were initially enrolled in the inaugural year of CPEM, from July 2018 to June 2019, including 11 in CPEM-Clinical (CPEM-C) and 21 in CPEM-Didactic (CPEM-D). CPEM-C participants consisted of unspecialized medical officers (MOs) or specialty-trained registrars practicing only in the IHHN ED (two Internal Medicine, one General Surgery). CPEM-D participants were either MOs (12), residents (6), and/or (non-EM) specialty trained ED supervisors (2) in one of five other hospitals in Karachi (Aga Khan University Hospital, Patel Hospital, Jinnah Post Graduate Medical Center, Shaheed Mohtarma Benazir Bhutto Trauma Center, and Imam Clinic), as well as one physician from the Pediatric ED of IHHN. All learners had at least a minimum of two years’ experience working in an ED as MOs. Candidates underwent an application and interview process prior to being selected for the program. In addition to the weekly sessions, CPEM- C participants also had access to clinical mentorship by international faculty during the other days of the week, and they completed case and procedure logs.

Instruction in the program was provided by both visiting international and local faculty. Visiting international faculty were composed of EM specialists who provided clinical mentorship (to CPEM-C learners only) and facilitated the weekly conferences for both groups over the course of two to four weeks. The inaugural year featured twelve visiting international faculty from Australia, Malawi, Saudi Arabia, the UK, and the US. Visiting international faculty completed an application and interview process and an orientation session with program leads in order to be selected for the position. Local EM faculty from within the host institution as well as other local hospitals also provided regular training. Instruction in the program was primarily conducted in English.

In this work, we assess the impact of this program on the clinical metrics and outcomes of patients seen at the Indus Hospital and Health Network (IHHN) in Karachi, Pakistan by evaluating outcomes before and after the program and by comparing the outcomes of patients seen by CPEM graduates and other physician groups at IHHN. We use previously described metrics adapted to this context that evaluate structure, process, and outcome to evaluate quality of care [9,10,11,12,13,14,15].

## Methods

### The Indus Hospital and Health Network (IHHN)

#### Overview

IHHN in Karachi is currently a 305-bed (240 inpatient beds, 65 day care beds) tertiary care facility, with a 26-bed Adult ED, six-bed Intensive Care Unit, six-bed Coronary Care Unit, and five Operating Rooms, serving a low-income catchment population in the Korangi District in Karachi, Pakistan. The ED can see more than 500 patients daily and has expanded from an initial 11 beds to 26 beds. Acuity level is designated at triage as P1 (most acute) to P5 (least acute), according to the Manchester Triage Score [17,18].

#### Population

Patients 14 years and one day of age and older are usually seen in the Adult ED (with the exception of pediatric oncology patients, who may be seen in the Pediatric Oncology ED up to age 18). To avoid any overlap with pediatric patients, all patients aged 15 and above July to December 2017 and July to December 2019 were included in the analysis. Sex was designated as male or female in the electronic health record (EHR). All patients meeting these criteria seen in the Adult ED in the immediate six months after the implementation of the program were included and matched with the same months just prior to the implementation of the program so as to match the seasonal variability in certain pathologies, such as infectious diseases.

### Clinical Metrics and Outcomes

Two sets of measures were used to evaluate for differences in the quality of care before and after CPEM training as well as between CPEM graduates and other physician groups. First, we used outcome and process measures, including mortality, length of stay, and time-to-evaluation. Second, we used or estimated quality metrics for the care of patients with suspected cardiovascular disease/acute coronary syndrome, sepsis, respiratory disease, and trauma based on those previously described in the literature and adapted to the setting and available data from the EHR at IHHN [9,10,11,12,13,14,15]. As no standardized chief complaint data was available in 2017, we used vital signs and other measures to identify patients at risk for cardiovascular disease/acute coronary syndrome, sepsis, and respiratory disease (Table 2).

**Table 2:** Clinical metrics and outcomes used pre-/post-CPEM implementation. Patients at risk for cardiovascular disease/acute coronary syndrome, sepsis, respiratory disease, and trauma were identified using these parameters and metrics based on prior studies and measures of high-quality emergency care in international guidelines and standards. *ECG: electrocardiogram; FAST: Focused Assessment with Sonography in Trauma*.


	POPULATION	METRIC

	All	Mortality

	All	Length of stay (LOS)

	All	Time-to-evaluation

**Risk for Cardiovascular Disease/Acute Coronary Syndrome**	Patients ≥40	Rate of ECG and troponin ordering as well as repeat testing [19,20,21,22,23]

**Risk for Sepsis**	Patients with a shock index of >0.9 and fever (100.4°F) [19,24,25]	Rate of lactate and blood culture ordering[19,24,25]

**Risk for Respiratory Disease**	Patients with hypoxia (pulse oximetry saturation ≤92%) [19,26]	Rate of chest x-ray ordering

**Risk for Intra-Abdominal Trauma**	Patients with chief complaint including trauma, assault, injury, or fall	Rate of FAST exam completion [19,27]


For mortality, patients are, unfortunately, often brought to the ED after expiring at home some time before presentation. For this study, patients with a diagnosis of “brought dead” without any recorded ordered interventions in the record and/or with a length of stay (LOS) less than 10 minutes were not included in the mortality rate.

As no standardized chief complaint data were available for patients in 2017, and given the high incidence of cardiovascular disease in the Pakistani population, the rate of electrocardiogram (ECG) and troponin ordering in all patients ≥40 years of age was used to evaluate the quality of care for patients at risk for cardiovascular disease/acute coronary syndrome (ACS) [19,20,21,22,23].

For patients potentially at risk for sepsis, the rate of lactate and blood culture ordering in all patients with a shock index (defined as heart rate divided by systolic blood pressure) >0.9 with a fever (defined as at temperature ≥100.4°F) was used.

For patients potentially at risk for respiratory disease, the rate of chest x-ray ordering in patients with hypoxia in triage (defined as a pulse oximetry measurement of 92% or less) was used [19,26].

Patients potentially at risk for intra-abdominal trauma were identified using records indicating “injury,” “trauma,” “assault,” and/or “fall”—key words which were found to encompass both blunt and penetrating trauma, including road traffic accidents (RTAs), and the rate of FAST (Focused Assessment with Sonography in Trauma) completion was used [19,27].

Statistical analyses (summary statistics, two-sample independent tests and tests of proportions, and multinomial logistic regression) were conducted using Microsoft Excel (Microsoft Office Professional Plus 2016, Microsoft Corporation, Redmond, Washington, USA), MATLAB (R2015a, The MathWorks, Inc., Natick, MA, USA), and Stata (Stata 17.0-BE, StataCorp, College Station, Texas, USA). The study was approved by the Institutional Review Board at IHHN (Protocol Number: IRD_IRB_2019_01_007). Informed consent was obtained by program participants and was not required for patients given data was obtained by record review and de-identified for analysis.

## Results

### Outcomes Before and After CPEM Implementation

A total of 126,437 patients 15 years and older were seen at IHHN from July to December 2017 and July to December 2019. An average of 300 patients were seen per day in 2017 and 387 per day in 2019. In 2019, patient volume was higher and patients were younger, more likely to be female, and have slightly lower acuity on average. Length of stay (LOS) (2017: 06:33, 2019: 08:21, p < 0.0001) and time-to-evaluation (2017: 00:48, 2019: 01:02, p < 0.0001) also increased significantly between the two years. Mortality was low in general; there was a small but non-significant decrease in mortality (19/10,000 patients in 2019 vs 20/10,000 patients in 2017; p = 0.7943). Between 2017 and 2019, all clinical metrics improved significantly (Table 3).

**Table 3:** Clinical outcomes and metrics pre- and post-CPEM. Two sample proportion tests and two sample t-tests were used to compare years. ECG: electrocardiogram; FAST: Focused Assessment with Sonography in Trauma. *Point-of-care ultrasound (i.e., the FAST exam) was not being performed in IHHN in 2017.


		2017	2019	p-VALUE

	**Population Total**	55,213	71,224	NA

	**Mean Age** (years)	38.84 (SD 16.94)	37.99 (SD 16.61)	p < 0.0001

	**Sex** (F:M)	0.83:1(25,051 females)	0.94:1(34,469 females)	p < 0.0001

	**Acuity Level**(Level P1-P5)	2.72 (SD 0.70)	2.85 (SD 0.79)	p < 0.0001

	**Mortality**	109 deaths(20/10,000 patients)	136 deaths(19/10,000 patients)	p = 0.7397

	**Length of Stay** (hh:mm)	06:33 (SD 05:40)	08:21 (SD 06:58)	p < 0.0001

	**Time-to-Evaluation** (hh:mm)	00:49 (SD 01:13)	01:02 (SD 01:23)	p < 0.0001

**Risk for Cardiovascular Disease/Acute Coronary Syndrome**	**Total at Risk** (% of Total Population)	23,797 (43%)	28,608 (40%)	p < 0.0001

	**ECG** (% of Total at Risk)	6,500 (27%)	8,056 (28%)	p = 0.0314

	**Repeat ECG** (% of Total with ECG)	925 (14%)	1,291 (16%)	p = 0.0027

	**Troponin** (% of Total at Risk)	3,155 (13%)	4,419 (15%)	p < 0.0001

	**Repeat Troponin** (% of Total with Troponin)	64 (2%)	266 (6%)	p < 0.0001

**Risk for Sepsis**	**Total at Risk** (% of Total Population)	3,872 (7%)	3,666 (5%)	p < 0.0001

	**Blood Cultures** (% of Total at Risk)	403 (10%)	894 (24%)	p < 0.0001

	**Lactate** (% of Total at Risk)	117 (3%)	301 (8%)	p < 0.0001

	**Blood Cultures and Lactate** (% of Total at Risk)	58 (1%)	114 (3%)	p < 0.0001

**Risk for Respiratory Disease**	**Total at Risk** (% of Total Population)	1,682 (3%)	2,023 (3%)	p < 0.0001

	**Chest X-ray** (% of Total at Risk)	953 (57%)	1,250 (62%)	p = 0.0015

**Risk for Intra-abdominal Trauma**	**Total at Risk** (% of Total Population)	NA*	2,587 (4%)	NA
	
	**FAST Exam** (% of Total at Risk)		214 (8%)	


### Physician Group Analysis

We next evaluated the outcomes in CPEM graduates in 2019 compared to other trainees and non-trainees in the IHHN ED (n = 64,447 patient visits). A total of 6,777 visits in 2019 were excluded due to no provider being identified (9.5% of visits).

We first evaluated mortality between the two groups who most commonly staff the Resuscitation Bay (5 beds) in the Adult ED—CPEM graduates and other trainees in the ED (residents as well as the second cohort of CPEM trainees). There was no significant difference in mortality (p = 0.1478), though there was a trend toward decreased mortality in the group of CPEM graduates (26/10,000 patients versus 35/10,000 patients, p = 0.1478). Other clinical outcomes and metrics are shown in Table 4.

**Table 4:** Comparison of populations, outcomes, and metrics of CPEM graduates versus other trainees. Two sample proportion tests and two sample t-tests were used to compare cohorts. *ECG: electrocardiogram; FAST: Focused Assessment with Sonography in Trauma*.


		CPEM	OTHER TRAINEES	p-VALUE

	**Population Total**	12,394	20,242	NA

	**Mean Age** (years)	39.39 (SD 16.83)	39.52 (SD 17.20)	p = 0.5088

	**Sex** (F:M)	0.85:1(5699 female)	0.88:1(9478 female)	p = 0.1391

	**Acuity Level**(Level P1-P5)	2.74 (SD 0.78)	2.63 (SD 0.79)	p < 0.0001

	**Mortality**	32 deaths(26/10,000 patients)	71 deaths(35/10,000 patients)	p = 0.1478

	**Length of Stay** (hh:mm)	06:35 (SD 04:11)	05:46 (SD 04:38)	p < 0.0001

	**Time-to-Evaluation** (hh:mm)	01:06 (SD 01:15)	01:12 (SD 01:25)	p < 0.0001

**Risk for Cardiovascular Disease/Acute Coronary Syndrome**	**Total at Risk** (% of Total Population)	5,547 (45%)	9,054 (45%)	p = 0.9624

	**ECG** (% of Total at Risk)	1,691 (30%)	3,249 (36%)	p < 0.0001

	**Repeat ECG** (% of Total with ECG)	240 (14%)	545 (17%)	p = 0.0185

	**Troponin** (% of Total at Risk)	903 (16%)	1,852 (20%)	p < 0.0001

	**Repeat Troponin** (% of Total with Troponin)	58 (6%)	108 (6%)	p = 0.5402

**Risk for Sepsis**	**Total at Risk** (% of Total Population)	739 (6%)	1,371 (7%)	p = 0.0039

	**Blood Cultures** (% of Total at Risk)	140 (19%)	354 (26%)	p = 0.0004

	**Lactate** (% of Total at Risk)	69 (9%)	135 (10%)	p = 0.7054

	**Blood Cultures and Lactate** (% of Total at Risk)	22 (3%)	52 (4%)	p = 0.3311

**Risk for Respiratory Disease**	**Total at Risk** (% of Total Population)	483 (4%)	935 (5%)	p = 0.0019

	**Chest X-ray** (% of Total at Risk)	283 (59%)	588 (63%)	p = 0.1153

**Risk for Intra-abdominal Trauma**	**Total at Risk** (% of Total Population)	517 (4%)	949 (5%)	p = 0.0287

	**FAST Exam** (% of Total at Risk)	90 (17%)	178 (19%)	p = 0.5233


Next, we compared CPEM graduates to all other non-trainee staff in the ED. CPEM graduates saw fewer female patients, older patients, and patients of higher acuity on average. CPEM graduates had a shorter LOS, and either similar or improved metrics compared to non-trainees, except in the ordering of blood cultures in patients with a shock index >0.9 and a fever (Table 5).

**Table 5:** Comparison of populations, outcomes, and metrics of CPEM graduates versus non-trainees. Two sample proportion tests and two sample t-tests were used to compare the cohorts. *ECG: electrocardiogram; FAST: Focused Assessment with Sonography in Trauma*.


		CPEM	NON-TRAINEES	p-VALUE

	**Population Total**	12,394	31,811	N/A

	**Mean Age** (years)	39.39 (SD 16.83)	36.70 (SD 16.16)	p < 0.0001

	**Sex** (F:M)	0.85:1(5,699 female)	1.24:117,334 (female)	p < 0.0001

	**Acuity Level** (Level P1-P5)	2.74 (SD 0.78)	2.82 (SD 0.68)	p < 0.0001

	**Mortality**	32 deaths (26/10,000 patients)	33 deaths(10/10,000 patients)	p = 0.0001

	**Length of Stay** (hh:mm)	06:35 (SD 04:11)	06:50 (SD 05:40)	p < 0.0001

	**Time-to-Evaluation** (hh:mm)	01:06 (SD 01:15)	01:04 (SD 01:25)	p = 0.0662

**Risk for Cardiovascular Disease/Acute Coronary Syndrome**	**Total at Risk** (% of Total Population)	5,547 (45%)	11,351 (36%)	p < 0.0001

	**ECG** (% of Total at Risk)	1,691 (30%)	3,105 (27%)	p < 0.0001

	**Repeat ECG** (% of Total with ECG)	240 (14%)	503 (16%)	p = 0.0665

	**Troponin** (% of Total at Risk)	903 (16%)	1,660 (15%)	p = 0.0049

	**Repeat Troponin** (% of Total with Troponin)	58 (6%)	98 (6%)	p = 0.5993

**Risk for Sepsis**	**Total at Risk** (% of Total Population)	739 (6%)	1539 (5%)	p < 0.0001

	**Blood Cultures** (% of Total at Risk)	140 (19%)	400 (26%)	p = 0.0002

	**Lactate** (% of Total at Risk)	69 (9%)	97 (6%)	p = 0.0091

	**Blood Cultures and Lactate** (% of Total at Risk)	22 (3%)	40 (3%)	p = 0.5239

**Risk for Respiratory Disease**	**Total at Risk** (% of Total Population)	483 (4%)	593 (2%)	p < 0.0001

	**Chest X-ray** (% of Total at Risk)	283 (59%)	379 (63%)	p = 0.744

**Risk for Intra-abdominal Trauma**	**Total at Risk** (% of Total Population)	517 (4%)	1,042 (3%)	p < 0.0001

	**FAST Exam** (% of Total at Risk)	90 (17%)	58 (7%)	p < 0.0001


### Additional Outcomes

Using multinomial logistic regression, the relative risk of death was 0.587 in 2019 compared to 2017, accounting for age, sex, and acuity score (Table 6). There was no significant different in relative risk of death between CPEM graduates and other trainees and CPEM graduates and non-trainees, when accounting for age, sex, and acuity score (Table 6).

**Table 6:** Relative risk of death with age, sex, and acuity as covariates using multinomial logistic regression.


PRE- AND POST-IMPLEMENTATION OF CPEM			

Risk of Year (2019, relative to 2017)	0.587	0.452–0.763	p < 0.0001

**PHYSICIAN GROUP ANALYSIS**			

Risk of CPEM Graduates versus Other Trainees	0.844	0.553–1.289	p = 0.432

Risk of CPEM Graduates versus Non-Trainees	1.298	0.783–2.154	p = 0.312


## Discussion

This study, overall, shows improvements in clinical metrics and outcomes after the implementation of a novel, intensive one-year certification program in EM in Karachi, Pakistan. Mortality rates before and after implementation, and when comparing CPEM graduates to other trainees, trended toward improvement, but were not statistically significant, and likely related to the overall low mortality rate in IHHN ED. Most notably, when comparing CPEM to non-trainees (excluding not fully trained CPEM trainees, residents, and faculty), CPEM graduates saw older and higher acuity patients and with a shorter LOS, despite the need for additional clinical evaluation and testing. Additionally, their performance on the identified metrics were, overall, improved compared to non-trainees.

We included the comparisons of CPEM trainees to other trainees and non-trainees in the ED in order to evaluate the potential similarities and/or differences of the graduates of the program as compared to the other provider groups in the ED. When comparing CPEM graduates to other trainees (residents and CPEM trainees in the first six months of training)—who were found to have a more comparable patient population with CPEM graduates than non-trainees (e.g., no significant difference in age or sex)—CPEM graduates did not significantly differ in the majority of metrics (repeat troponin, lactate, blood cultures and lactate, chest x-ray, and FAST exam); in all other metrics, the other trainees did out-perform the CPEM graduates. Although a more comparable patient population, the current trainees did still see slightly higher acuity patients than the CPEM graduates, which may explain this difference. Also, the second cohort of CPEM (included in the other trainee group) was actively enrolled in the course and going through the Cardiovascular, Pulmonary, and Trauma blocks during the study period, which address the majority of metrics used in this study. Interestingly, the other trainees had a shorter length of stay than the CPEM graduates, despite tending to order more tests, which has not been shown to be the case in other studies [28]. It is possible these patients were admitted or referred out to other hospitals more quickly due to their higher acuity.

The data did show a significantly lower relative risk of death of 0.587 (p < 0.001) in 2019 as compared to 2017, accounting for acuity, sex, and age, with the CPEM program being the primary project implemented during that time. CPEM graduates did have a lower relative risk of death compared to other trainees F(0.884, p = 0.432), but slightly higher than non-trainees (1.298, p = 0.312), although the results were not significant. Again, although this multinomial regression accounts for acuity level, trainees are usually included in managing the most acute cases and codes, which may partially account for these results.

Of note, the CPEM program is not meant to replace existing training pathways and structures but bridge the gap in training as these programs further develop. These results suggest that CPEM graduates are on par with early level trainees. Another aim of the program is to improve employee retention and to encourage specialization in EM through residency training. Twenty-five of the original 32 CPEM enrollees (78.1%) completed the certificate program (9 of 11 CPEM-C learners, 16 of 21 CPEM-D learners) in the initial cohort. By the end of Dec 2019, eight of the CPEM-C graduates were still working in the Indus ED, and one CPEM-D candidate had started working at IHHN.

To date, few evaluations of clinical metrics and outcomes after educational initiatives have been conducted; however, our results do correspond to other previously published studies [29,30,31]. For example, Aluisio and co-authors demonstrated a substantial decrease in Emergency Center (EC) mortality (6.3% to 1.2%) in Rwanda after the implementation of a post-graduate diploma program, although the diploma program in this setting transitioned into a four-year residency program during the study and other interventions, such as formalized triage, team-based resuscitation, and the designation of higher acuity areas also occurred in the EC during the study period, likely contributing to the significant decrease in mortality [29].

There are several limitations of this study. We used some standards of care and/or metrics mostly studied in higher-resource settings; quality metrics are not always linked to patient outcomes; and little is known if these are also appropriate for lower-resource settings [9,10,11,12,13,14,15,32,33]. Additionally, we adapted these metrics to the data available. As a result, we chose to identify patients that might be ‘at risk’ for or suspected as having certain pathologies, rather than those with final, confirmed diagnoses. Further research is needed to define and validate key metrics and outcomes that will allow for further development of emergency care systems research to improve the quality of care for patients presenting to EDs with emergency conditions, particularly in low-resource settings [9,10,11,32,33].

The data available in the EHR also introduced some additional limitations. Age, vital signs, and other measures were used to identify patients “at risk” for cardiovascular disease, sepsis, respiratory disease, and intra-abdominal trauma, both to identify patients more objectively and because of non-standardization of the coding for presenting complaints, physician notes, and final diagnosis. At the time of the study, only non-medication orders were able to be linked to patient visits. Additionally, timings in the EHR are recorded when a physician note is opened, which may be delayed due to the need to stabilize the patient, which could affect the accuracy of time-to-evaluation times, although this is expected to be similar in all cohorts as there is no specific training in the CPEM program or among other trainees regarding the timing of opening a note of which we are aware. A similar limitation was noted in LOS, where patients who are not manually discharged from the system are automatically removed after roughly 24 hours, though, again, this should be similar in all cohorts.

Finally, in the post-implementation analysis, a large number of the encounters did not have a physician identified, resulting in exclusion of this data. Partially as a result of this study, efforts are underway at IHHN to advance the process of data collection in the ED in order to make more targeted improvements in education as well as in clinical policies and guidelines.

Additionally, the overall results before and after the implementation of CPEM may have been affected by the start of the second cohort of CPEM as well as residency training in EM started during the post-analysis and between the pre-/post-analysis respectively; hence the analysis by physician group in the post-implementation period. Of note, only four residents started working in the ED in 2018 and five in 2019. Generally, residents spend a total of only six months in the adult ED over their first two years of training.

Overall, this study suggests that a medium duration EM training program may improve clinical metrics and outcomes and help bridge the gap in training, particularly in low-resource settings and in environments where EM is still an emerging specialty. More research is needed to identify additional metrics and outcomes, to link metrics to outcomes, and to validate these results with the implementation of similar programs in other settings.
